# Secondary findings and carrier test frequencies in a large multiethnic sample

**DOI:** 10.1186/s13073-015-0171-1

**Published:** 2015-06-13

**Authors:** Tomasz Gambin, Shalini N. Jhangiani, Jennifer E. Below, Ian M. Campbell, Wojciech Wiszniewski, Donna M. Muzny, Jeffrey Staples, Alanna C. Morrison, Matthew N. Bainbridge, Samantha Penney, Amy L. McGuire, Richard A. Gibbs, James R. Lupski, Eric Boerwinkle

**Affiliations:** Department of Molecular and Human Genetics, Baylor College of Medicine, Houston, TX 77030 USA; Institute of Computer Science, Warsaw University of Technology, Warsaw, 00-665 Poland; The Human Genome Sequencing Center, Baylor College of Medicine, Houston, TX 77030 USA; Human Genetics Center, University of Texas Health Science Center at Houston, Houston, TX 77030 USA; Department of Pediatrics, Baylor College of Medicine, Houston, TX 77030 USA; Texas Children’s Hospital, Houston, TX 77030 USA; Center for Medical Ethics and Health Policy, Baylor College of Medicine, Houston, TX 77030 USA

## Abstract

**Background:**

Besides its growing importance in clinical diagnostics and understanding the genetic basis of Mendelian and complex diseases, whole exome sequencing (WES) is a rich source of additional information of potential clinical utility for physicians, patients and their families. We analyzed the frequency and nature of single nucleotide variants (SNVs) considered secondary findings and recessive disease allele carrier status in the exomes of 8554 individuals from a large, randomly sampled cohort study and 2514 patients from a study of presumed Mendelian disease having undergone WES.

**Methods:**

We used the same sequencing platform and data processing pipeline to analyze all samples and characterized the distributions of reported pathogenic (ClinVar, Human Gene Mutation Database (HGMD)) and predicted deleterious variants in the pre-specified American College of Medical Genetics and Genomics (ACMG) secondary findings and recessive disease genes in different ethnic groups.

**Results:**

In the 56 ACMG secondary findings genes, the average number of predicted deleterious variants per individual was 0.74, and the mean number of ClinVar reported pathogenic variants was 0.06. We observed an average of 10 deleterious and 0.78 ClinVar reported pathogenic variants per individual in 1423 autosomal recessive disease genes. By repeatedly sampling pairs of exomes, 0.5 % of the randomly generated couples were at 25 % risk of having an affected offspring for an autosomal recessive disorder based on the ClinVar variants.

**Conclusions:**

By investigating reported pathogenic and novel, predicted deleterious variants we estimated the lower and upper limits of the population fraction for which exome sequencing may reveal additional medically relevant information. We suggest that the observed wide range for the lower and upper limits of these frequency numbers will be gradually reduced due to improvement in classification databases and prediction algorithms.

**Electronic supplementary material:**

The online version of this article (doi:10.1186/s13073-015-0171-1) contains supplementary material, which is available to authorized users.

## Background

Exome and genome sequencing is becoming an integral part of health care. Their role as molecular diagnostic tools in obstetrics [1] and pediatrics [2] is firmly established, as is their potential in hereditary cancer [3] and somatic testing [4]. Less well touted, but likely of broader application, is the use of sequencing in carrier testing for recessive disorders, as a subclinical marker of potential disease susceptibility or undiagnosed disease, and the development of genetic risk scores [5] to identify high risk individuals for a number of common chronic diseases. Like any test or procedure, DNA sequencing is able to detect findings for conditions other than the primary reason for which the original test was performed. These findings can be broadly divided into two groups. First, so-called secondary findings (SFs) [6, 7], i.e., variants in genes not directly related to the primary clinical diagnosis but actively screened due to their clinical importance, can have a direct impact on the health of the ascertained individual or family members with the same genotype. Second, sequence analysis can identify heterozygous alleles that have no obvious clinical manifestation in the carrier state but may impact future generations and reproduction decisions.

Previous reports of SF rates vary widely and generally focus on individuals highly ascertained on specific phenotypes and studies of relatively small sample sizes. Based on available data, it is expected that one to two percent of individuals will have at least one of the well-studied pathogenic variants originally identified in the American College of Medical Genetics and Genomics (ACMG) guidelines [8]. For example, Dorschner et al. [9] report the frequency of actionable pathogenic variants in 114 genes to be 3.4 % in European-descent and 1.2 % in African-descent individuals ascertained to be part of multiple case–control studies. To our knowledge no study has reported the rates of SFs in a large (e.g., >1,000 individuals) sample of individuals randomly selected from the US population. Further, the distortion of SF frequencies in regions populated with individuals with different specific continental origins is largely unexplored.

We identified both SFs and recessive carrier alleles in a large random sample of African-Americans (AAs; *N* = 2836) and European-Americans (EAs; *N* = 5718) from the US population. We also ascertained European (*N* = 1455), African (*N* = 122) Turkish (*N* = 498), Hispanic (*N* = 388) and Asian (*N* = 51) samples that were part of systematic studies to discover novel Mendelian disease genes. We utilized the same platform and algorithms for all samples analyzed in this study, and were therefore able to compare the rates in the AA/EA populations with those in the other groups. By including information from multiple data sources ranging from the widely agreed upon and adjudicated variants in ClinVar [10] to predicted deleterious variants using dbNSFP [11], we were able to establish the lower and upper bounds, respectively, of both the SFs and recessive carrier alleles in diverse populations. The data presented here enable assessment of the impact of a comprehensive carrier testing program for established recessive disorders, keeping in mind the ever-changing nature of the reference databases, such as ClinVar and dbNSFP.

## Methods

This research conforms to the Helsinki Declaration and was approved by local institutional review boards. All study participants provided written informed consent and agreed to participate in genetic studies. Genetic studies in the Atherosclerosis Risk in Communities (ARIC) study have been approved by the Institutional Review Board at the University of Texas Health Science Center at Houston. Genetic studies in the Baylor-Johns Hopkins Center for Mendelian Genomics (CMG) have been approved by the Institutional Review Board at the Baylor College of Medicine at Houston. The data are available from dbGAP under the following accession numbers: ARIC phs000668.v1.p1, and Baylor Hopkins Center for Mendelian Genomics (CMG) phs000711.v2.p1.

### Cohorts

Whole exome sequencing was performed on 8554 individuals derived from the Atherosclerosis Risk in Communities study [12] (ARIC) and from 2514 patients sequenced at the CMG at Baylor College of Medicine [13]. As part of ongoing efforts to identify genes influencing risk of common heart, lung and blood diseases, we are performing exome sequencing on members of the ARIC study. A total of 15,792 individuals, predominantly EA and AA, participated in the ARIC study baseline examination in 1987–1989, with three additional triennial follow-up examinations and a fifth exam in 2011–2013. The ARIC cohort includes a sample of individuals aged 45–64 years randomly selected and recruited from four US communities: suburban Minneapolis, MN; Washington County, MD; Forsyth County, NC; and Jackson, MS [12]. All individuals whose data are included here provided written informed consent for large-scale genomic studies and broad data sharing. Ethnic classification of the ARIC study sample was confirmed with principal components analysis performed using the EIGENSTRAT software [14].

The primary goal of the CMG is to identify novel genes responsible for Mendelian conditions [13]. CMG study participants are heterogeneous in terms of phenotypic presentation and ethnic origins. The total number of distinct Mendelian conditions representing clinical diagnoses included in this sample set was 250 (Additional file 1). Samples were collected from 23 countries from North and South Americas, Europe, Asia and Australia. To obtain unbiased ethnic classification of the CMG study participants, we used PRIMUS [15] and genotype data from Illumina’s Human Exome (v.1-1 or v.1-2) arrays. PRIMUS encapsulates the upstream quality control (QC) required before principal components analysis and uses a clustering algorithm to assign ancestral groups to the samples using principal components derived from the EIGENSTRAT software [14].

### Sequencing and QC

DNA samples were processed according to protocols previously described [16]. Sequencing was performed using Illumina Hi-Seq (San Diego, CA) instruments after exome capture with the Baylor Human Genome Sequencing Center VCRome 2.1 (ARIC samples) or CORE [17] (CMG samples) designs. To minimize the influence of differences between the two designs on the results of the comparative analysis, we identified the intersection of the capture designs and excluded variants located outside the regions of overlap. Raw sequence data were post-processed using the Mercury pipeline [18]. The Mercury pipeline performs conversion of raw sequencing data (bcl files) to a fastq format using Casava, mapping of the short reads against a human genome reference sequence (GRCh37) using the Burrows-Wheeler Alignment (BWA), recalibration using GATK [19], and variant calling using the Atlas2 suite [20]. Finally, Cassandra [21] was used to annotate relevant information about gene names, predicted variant pathogenicity, reference allele frequencies and metadata from external resources, and then to add these to the Variant Call Format (VCF) file.

After initial data processing every sample was evaluated using rigorous QC metrics, including percentage of targets covered at 20× or greater and concordance of single nucleotide polymorphisms (SNPs) calls between exome sequencing and SNP array data. Additionally, each SNP variant call was filtered using the following criteria: low single nucleotide variant (SNV) posterior probability (<0.95), strand-bias of more than 99 % variant reads in a single strand direction and total coverage less than tenfold. Moreover, sample level QC for ARIC cohort removed known and blind duplicates, samples with known sex mismatches (indicating sample contamination), samples with missing rate >65 % and extreme outliers (e.g., singleton counts). Only samples that passed QC were included in this analysis.

### Variants filtering

From the variants obtained by exome sequencing, we selected nonsynonymous variants in a prespecified list of 56 SF genes or 1423 autosomal recessive disorder genes. Additionally, for the analysis of females, we selected nonsynonymous variants in an additional set of 112 X-linked recessive genes. The list of SF genes was obtained from the ACMG recommendations for reporting of secondary findings in clinical exomes [8], and includes 56 genes associated with 24 conditions, most of which are inherited dominantly. The list of autosomal recessive genes was created based on an extensive search of the MedGen database [22], for all autosomal recessive disorders (see Additional file 2 for details of gene extraction procedure). The initial set of 1496 genes obtained from MedGen was compared with the lists of autosomal recessive disease genes described in previous studies [23, 24] (Additional file 3). Next, we manually evaluated Online Mendelian Inheritance in Man (OMIM) entries for 314 MedGen genes not reported in the previous studies to confirm that these genes are truly associated with the autosomal recessive disorders. We were able to identify a corresponding OMIM entry for each of the 314 genes. Of those, we excluded 72 genes in which we did not find evidence of homozygous or compound heterozygous variants causing Mendelian disease. We excluded *TTN*, which is the most commonly mutated gene and could have an exaggerated influence on these results. We present the list of genes at each filtering step in Additional file 4. The final list of 1423 genes associated with 1493 disorders is presented in Additional file 5. Similarly, the list of 112 genes associated with 159 X-linked recessive disorders (Additional file 6) was obtained using the MedGen database and then manually curated. Since the number of genes was significantly smaller than in the case of autosomal recessive disease genes, we evaluated all of these genes in OMIM without comparing them with the lists from previous studies. From the original list of the 126 genes extracted from MedGen, we excluded genes for which we did not find evidence that a hemizygous variant in a male is associated with a Mendelian condition (Additional file 7).

To establish an upper bound for the frequency of potential secondary findings, we considered the list of stop-gain (nonsense), stop-loss and missense variants predicted to be deleterious by the RadialSVM algorithm [11, 25]. We excluded variants with minor allele frequency (MAF) >1 % in control databases (Exome Sequencing Project [ESP] and 1000 Genomes) or with MAF >2 % in our cohort and variants of low quality, i.e., with depth of coverage <20 and/or with the ratio of variant reads to total reads <0.2. The list of predicted deleterious variants was determined using the RadialSVM score provided in dbNSFP v.2.5 [11, 25]. This support vector machine (SVM)-based ensemble prediction score incorporates ten other scores (SIFT, PolyPhen-2 HDIV, PolyPhen-2 HVAR, GERP++, MutationTaster, Mutation Assessor, FATHMM, LRT, SiPhy, PhyloP) and the maximum allele frequency observed in the 1000 Genomes populations [11]. In comparison studies [26], this method was shown to outperform other prediction algorithms with the highest Mathews correlation coefficient (0.474) and relatively low false negative rate (5 %) and false positive rate (57 %). RadialSVM was applied to all rare variants regardless of their classification in HGMD or ClinVar. We removed nonsense variants that are located in the last exon or in the last 50 bp of the penultimate exon, which are likely to escape nonsense-mediated decay (NMD) and thus they may be less damaging [27].

Reported pathogenic variants were obtained from the ClinVar [10] and HGMD (Professional version 2012.4) [28] databases. In this analysis, we considered a HGMD variant as reported pathogenic if it was annotated as “Disease-causing Mutation” (DM).

The ClinVar data used in this analysis were extracted from the “clinvar-latest.vcf” file generated on 4 June 2014. Variants in ClinVar are reported by single or multiple submitters, which may result in discordant classifications. In this study, we defined a variant as “pathogenic” if: (i) no submitter reported this variant as “benign” or “likely benign”; and (ii) at least one submitter classified this variant as “pathogenic”. We did not include variants classified as “likely pathogenic” unless another submitter reported them as “pathogenic”. We did not use the recently implemented star rating in this analysis.

### Number of ClinVar submissions for different ethnic groups

We performed a comparison of the number of ClinVar submission entries among four populations, including Europeans, Africans, Hispanics and Asians (see Additional file 2 for details).

## Results

**Fig. 1:**
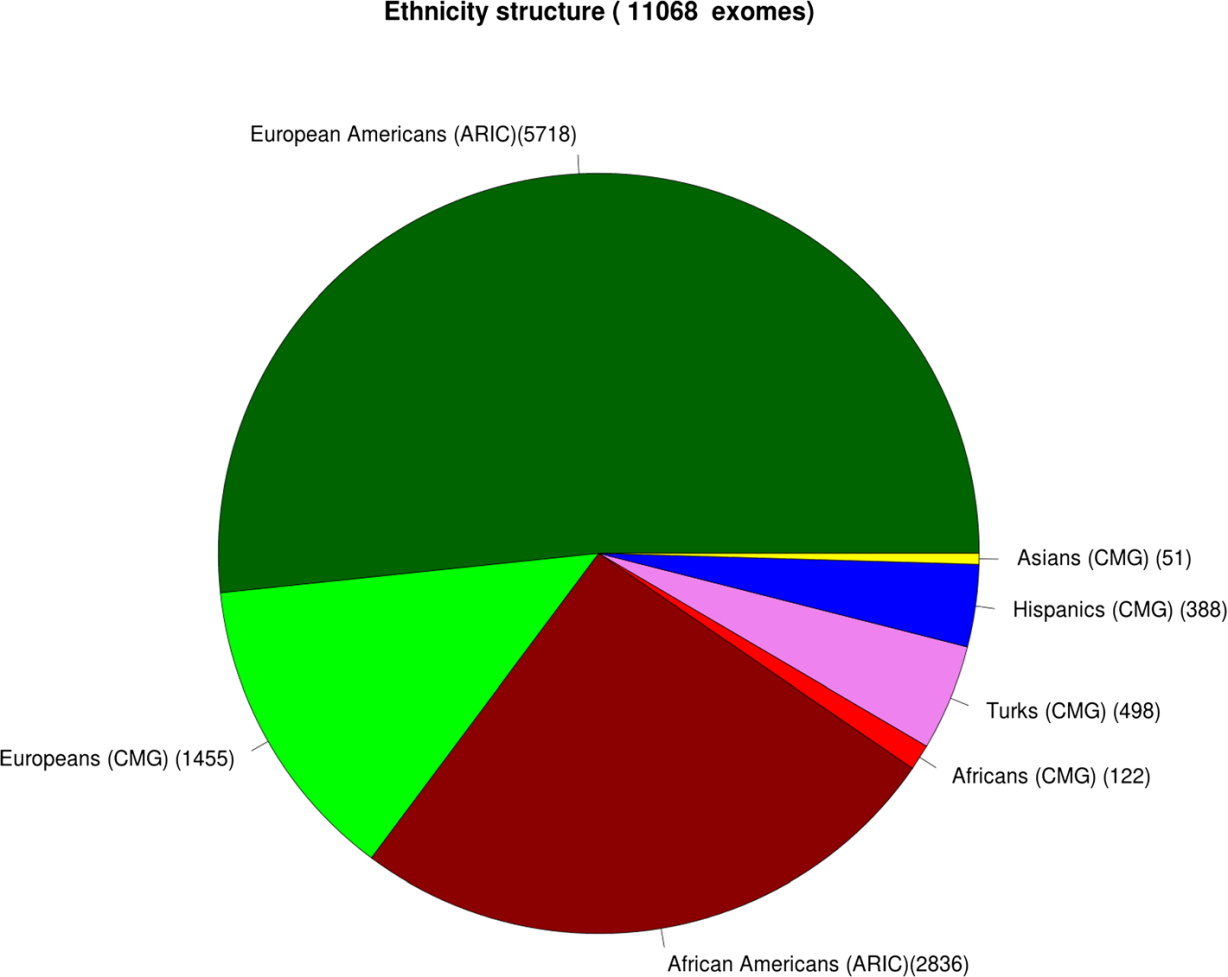
Assigned ethnicity by study origin for 8554 ARIC and 2514 CMG individuals

After QC, 8554 exomes were available from the ARIC cohort and 2514 exomes were available from the CMG. The average coverage was 92× and 105×, respectively. We calculated coverage for the genes considered in this analysis and the average percentage of bases with coverage of 20× or greater was 95 % for both ARIC and CMG. ARIC included 5718 and 2836 self-reported EAs and AAs, respectively. The average numbers of variants per individual with a MAF <5 % were 1765 and 3870 for ARIC EAs and AAs, respectively. The CMG sample set is more ethnically heterogeneous resulting from the overall objective of the program and the global nature of their ascertainment. Additional file 8 shows the distributions of the first two principal components relative to HapMap comparison groups. A pie diagram of the assigned ethnic group and study (ARIC or CMG) is presented as Fig. 1.

### Reported pathogenic variants in SF genes

**Fig. 2:**
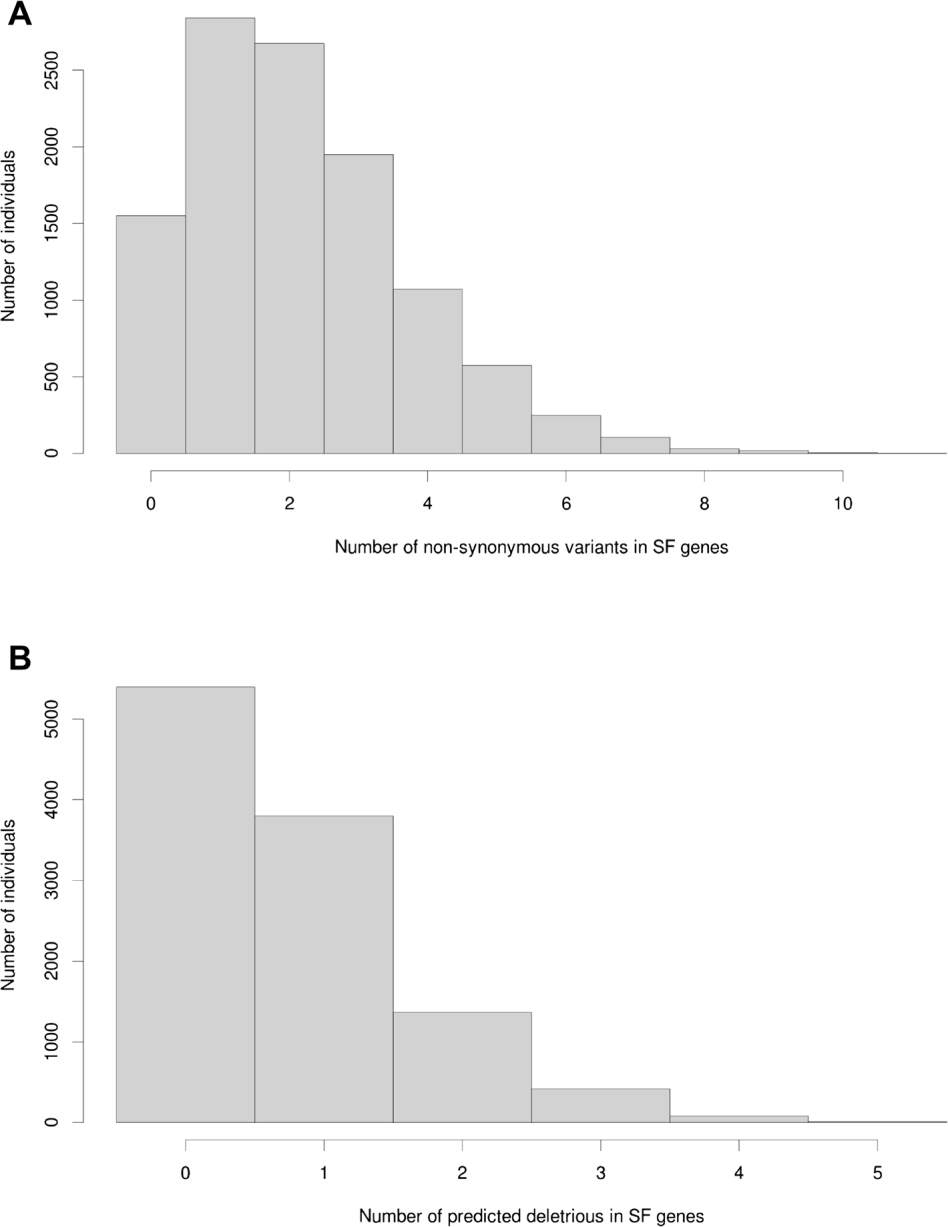
Distribution of the number of annotated variants per individual in 56 ACMG SF genes. **a** Rare nonsynonymous variants. **b** Predicted deleterious variants

In the 11,068 exomes from ARIC and the CMG, 6221 unique nonsynonymous variants in the 56 ACMG SF genes had a MAF of less than 1 %, and these variants occurred 23,892 times across the study sample. The number of SF gene variants in an individual ranged from 0–11, with an average of 2.2 (median of 2) variants per individual. In 1550 individuals (~14 %), there were no nonsynonymous variants in any of the *a priori* identified SF genes, and six individuals had 10 or 11 such variants (Fig. 2). More than half of the variants (3831 out of 6221) were observed only once and, of those, 3091 variants were absent in the 1000 Genomes and ESP databases. Out of the 6221 nonsynonymous variants, we identified 2815 predicted deleterious variants occurring 8167 times. These variants were found in 51 % of individuals (5674 out of 11,068) with an average of 0.74 (median 1) predicted deleterious variants in the SF genes per individual (Fig. 2).

**Fig. 3:**
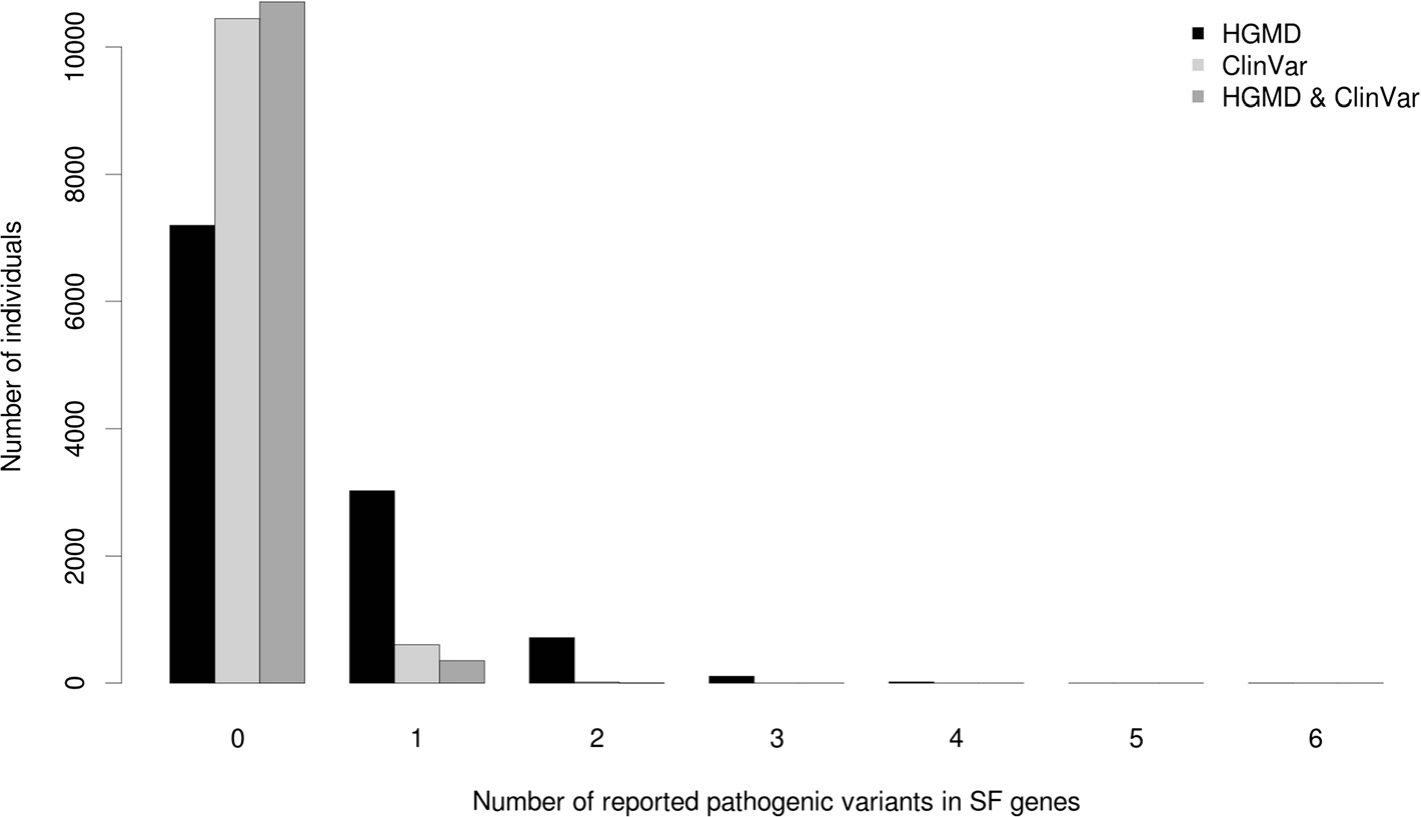
Distribution of the number of reported pathogenic variants per individual in 56 ACMG SF genes according to HGMD-DM (*black bars*), ClinVar (*light gray bars*) and combined (*dark gray bars*) databases

In the 56 ACMG SF genes, we observed 642 occurrences of 136 unique variants reported in ClinVar as pathogenic. These variants were present in 5.6 % of study samples (623 out of 11,068 individuals); 19 individuals had more than one ClinVar reported pathogenic variant (Fig. 3). Considering the HGMD-Disease-causing Mutation (HGMD-DM) categorization, approximately 10 % of all unique variants (645 out of 6221) were reported as pathogenic. Analysis of the distribution of HGMD-DM variants showed that 35 % of individuals (3871 out of 11,068) have at least one DM variant and of those 847 have two or more (Fig. 3).

Nonsense variants in SF genes were found in 2 % of the study sample (243 out of 11,068). Out of 76 unique nonsense variants, 13 were reported as pathogenic by both ClinVar and HGMD; one variant was reported only by ClinVar and 18 only by HGMD. We detected 40 novel nonsense variants (53 % of all nonsense variants) in the SF genes not classified in ClinVar or HGMD. After excluding variants located in NMD-escaping regions, we observed 30 (32 occurrences) rare nonsense variants in the SF genes that are likely to be pathogenic.

### Carrier detection

**Fig. 4:**
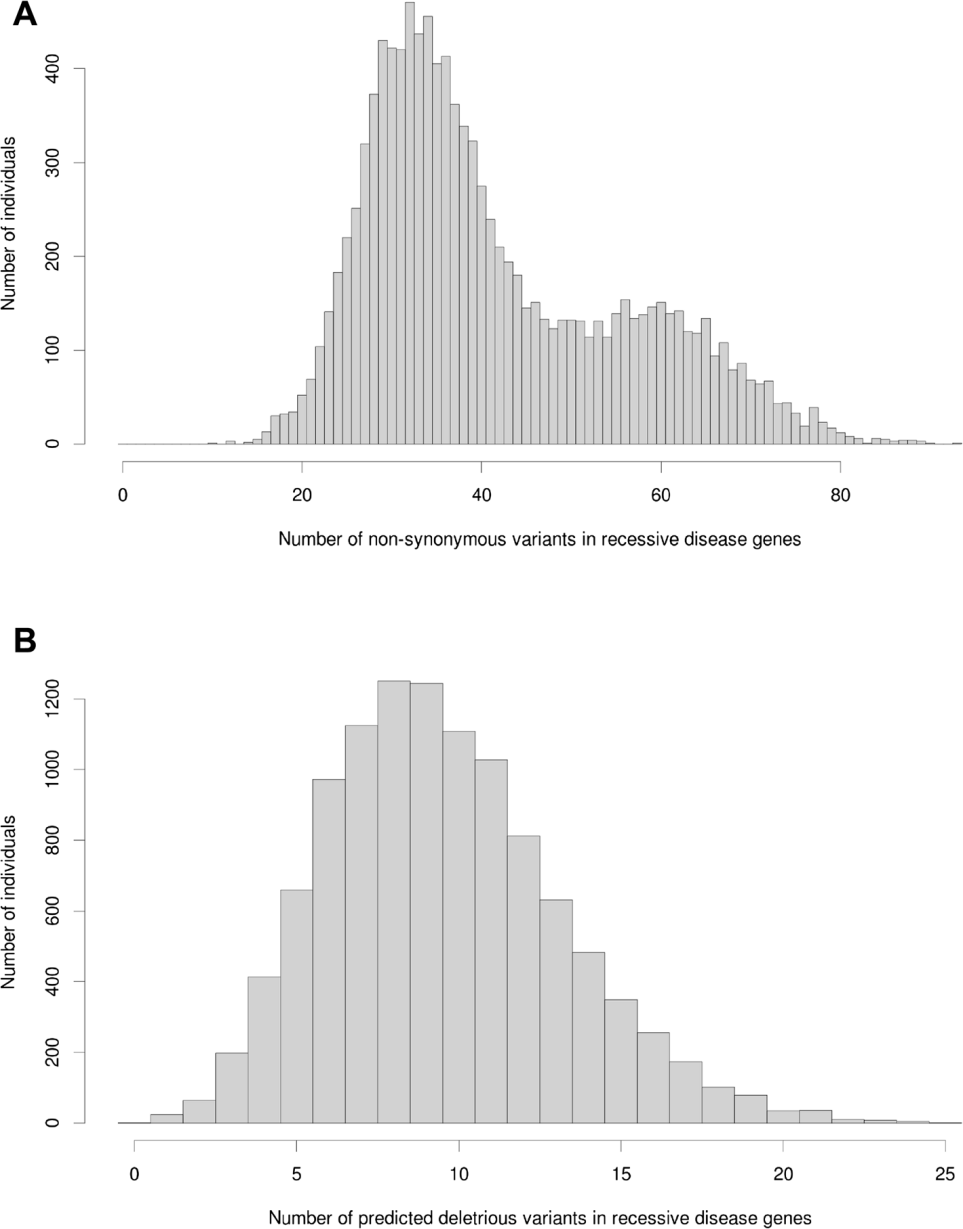
Distribution of the number of variants per individual in autosomal recessive disease genes. **a** Rare nonsynonymous variants. **b** Predicted deleterious variants

We identified 111,049 rare nonsynonymous variants in the a priori defined list of 1423 autosomal recessive disease genes. The frequency distribution of the number of autosomal recessive disease variants per individual is shown in Fig. 4 and appears bimodal, which reflects differences among ethnic groups, with EAs having lower numbers and AAs having higher numbers. After excluding non-deleterious variants, we observed 32,213 unique deleterious variants occurring 105,323 times. Individuals carried from 0–25 autosomal recessive disease variants with an average of ten (median nine), and one individual carried zero (Fig. 4). The majority of all variants (20,028 out of 32,213) were observed only once, and 16,106 of these were not reported in 1000 Genomes and ESP databases.

**Fig. 5:**
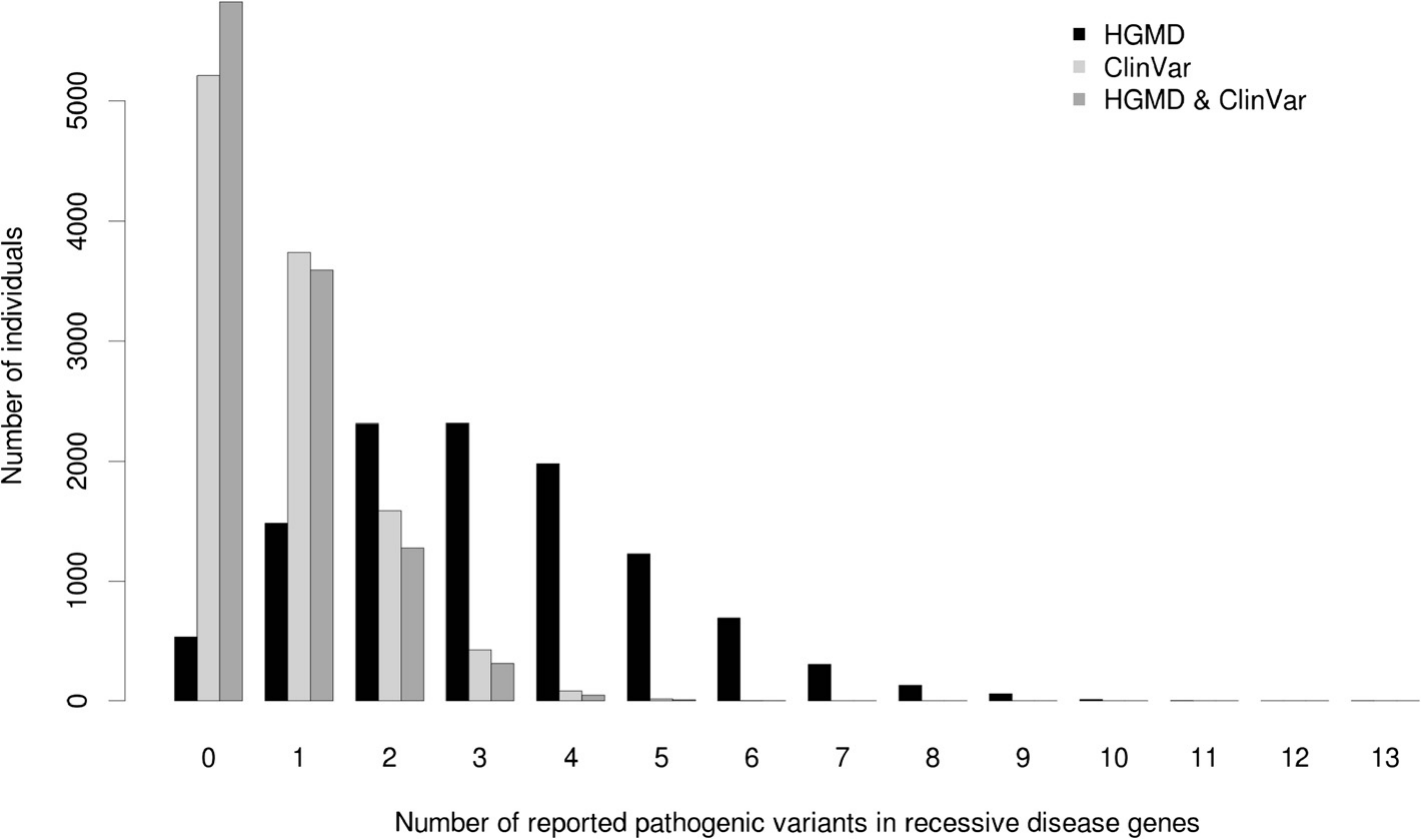
Distribution of the number of reported pathogenic variants per individual in autosomal recessive disease genes according to HGMD-DM (*black bars*), ClinVar (*light gray bars*), and combined (*dark gray bars*) databases

There were 1366 reported pathogenic ClinVar variants observed in the list of 1423 autosomal recessive disease genes, and these occurred 8634 times in the study sample. Fifty-three percent of individuals in the sample (5858 out of 11,068) carry at least one reported pathogenic ClinVar variant with an average of 0.78 (median = 1) variants per individual. Significantly more unique reported pathogenic variants (4435) were identified using HGMD-DM, and 95 % of the individuals (10,531 out of 11,068) contain at least one HGMD-DM variant in an autosomal recessive gene. The number of HGMD-DM variants in an individual ranged from 0 to 13 with an average of 3.2 (median of 3) per individual (Fig. 5). The majority of reported pathogenic ClinVar variants (1261 out of 1366) had a concordant annotation in HGMD. For these autosomal recessive genes, we also observed significantly higher MAFs of HGMD-DM variants (average MAF = 0.23 %; median MAF = 0.19 %) in comparison with the ClinVar variants (average MAF = 0.21 %; median MAF = 0.13 %) (Wilcoxon rank sum test, *p* value = 4.2e-33).

We found that 40 % of individuals (4164 out of 11,068) carry a nonsense variant in one of the autosomal recessive disease genes. From the total number of 2737 nonsense variants (5295 occurrences, average MAF = 0.06 %), 478 (1139 occurrences, average MAF = 0.09 %) were found in NMD-escaping regions, further supporting previous observations of a significantly higher average nonsense frequency in NMD-escaping regions than in other regions of a gene where they are expected to reduce gene expression [29, 30]. Approximately 10 % of nonsense variants in autosomal recessive genes (265 out of 2737) were reported as pathogenic by ClinVar and HGMD. Additionally, 393 variants were reported as pathogenic by only one of these databases (15 by ClinVar and 378 by HGMD). Seventy-five percent of the nonsense variants (2059 out of 2737) were not found in the 1000 Genomes and ESP databases. Out of those, 1667 (from 2705 occurrences) were located outside presumed NMD-escaping regions. These novel nonsense variants were identified in 22 % of all individuals (2380 out of 11,068).

### Percentage of couples at risk of having affected offspring

To estimate the percentage of couples in the general population in which both partners have a reported pathogenic variant in the same autosomal recessive disease gene, we performed the following resampling experiment using data from the ARIC study. From this random sample of individuals, which is likely representative of EA and AA couples planning to have children, we randomly sampled two exomes (one from a male and one from a female) to evaluate if they share at least one autosomal recessive disease gene with a reported pathogenic ClinVar variant. After 1,000,000 iterations, we observed that 0.5 % of couples are at risk of having an affected offspring. When this experiment was repeated using predicted deleterious variants in the same list of genes, the proportion of at-risk couples was 17.6 %, which forms a likely upper bound for the estimate. Additionally, we calculated that 5 % of females (241 out of 4817) are carriers of a ClinVar reported pathogenic variant in an X-linked recessive disease gene. A predicted deleterious variant in at least one X-linked disease gene was found in 33 % of females (1587 out of 4817).

### Comparison among ethnic groups

**Fig. 6:**
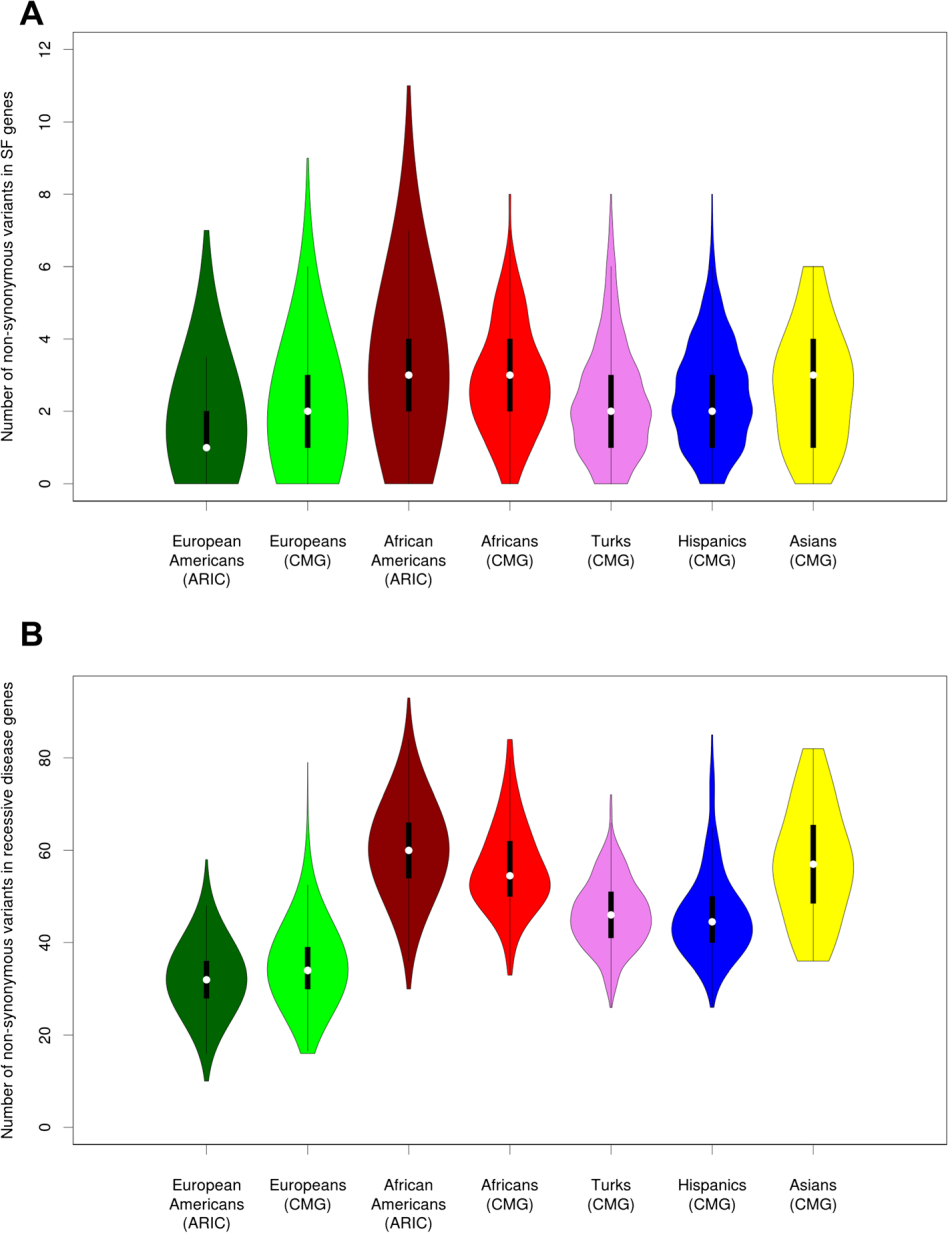
Distributions of the number of annotated nonsynonymous variants among ethnic groups in 56 ACMG SF genes (**a**) and in autosomal recessive disease genes (**b**)

To compare the total burden of alleles in the SF and autosomal recessive genes among five ethnic groups included in this study, we determined the average number of variants per individual and the fraction of individuals carrying at least one variant for each ethnic group (Table 1). AAs carry, on average, around three nonsynonymous variants in SF genes, and at least one SF variant was identified in 96 % of all AAs, whereas individuals from the other groups contain significantly fewer (Fig. 6). The fewest numbers of variants were found in individuals of European descent, where we observed that ~20 % of individuals do not have any nonsynonymous variants in SF genes. We did not observe significant differences in the number of reported pathogenic variants in ClinVar/HGMD among ethnic groups. Except in individuals of Asian descent where the sample size was small, 5–6 % of individuals in each group were carriers for at least one reported pathogenic variant according to ClinVar and 33–39 % in each group had an HGMD-DM variant.

**Table 1:** Average frequencies of variants in 56 ACMG SF genes among five ethnic groups

Population	European Americans (ARIC)	Europeans (CMG)	African Americans (ARIC)	Africans (CMG)	Turks (CMG)	Hispanics (CMG)	Asians (CMG)
Number of samples	5718	1455	2836	122	498	388	51
Average number of nonsynonymous variants per individual	1.683	1.878	3.159	2.852	2.271	2.472	2.667
Fraction of individuals with nonsynonymous variants	0.807	0.84	0.955	0.959	0.896	0.93	0.922
Average number of predicted deleterious variants per individual	0.612	0.737	0.963	0.779	0.865	0.765	0.843
Fraction of individuals with predicted deleterious variants	0.449	0.529	0.615	0.549	0.578	0.539	0.569
Average number of ClinVar variants per individual	0.06	0.049	0.06	0.057	0.056	0.044	0.137
Fraction of individuals with ClinVar variants	0.058	0.048	0.058	0.057	0.052	0.044	0.137
Average number of HGMD variants per individual	0.416	0.463	0.485	0.459	0.4	0.428	0.412
Fraction of individuals with HGMD-DM variants	0.335	0.355	0.38	0.393	0.333	0.34	0.353
Average number of nonsense variants per individual	0.007	0.006	0.013	0.008	0.008	0.003	0
Fraction of individuals with nonsense variants	0.007	0.006	0.013	0.008	0.008	0.003	0

These frequencies are reported for: 1) all rare nonsynonymous variants, 2) predicted deleterious variants, 3) reported pathogenic ClinVar variants, 4) HGMD-DM variants, 5) nonsense variants

Analysis of autosomal recessive genes showed that, on average, individuals of European ancestry carry from 32–35 nonsynonymous variants, whereas individuals of Hispanic (46), Turkish (46), Asian (57) and African descent (56–60) carry more (Table 2). An analogous pattern was observed for deleterious variants, but the relative differences among populations are slightly smaller. In contrast, the highest average number of reported pathogenic ClinVar variants was found in the European descent population (0.88–0.92), whereas the average in the African descent population was significantly smaller (0.49–0.53). The averages for other populations varied from 0.63 (Asians) and 0.67 (Hispanic) to 0.80 (Turkish). Similarly, the average number of HGMD-DM variants in individuals of European descent (3.44–3.59) was ~50 % higher than the average in those of African descent (2.23–2.35). Individuals with Turkish ancestry had the highest carrier frequency of HGMD-DM autosomal recessive alleles (3.85).

**Table 2:** Average frequencies of variants in autosomal recessive disease genes among five ethnic groups

Population	European Americans (ARIC)	Europeans (CMG)	African Americans (ARIC)	Africans (CMG)	Turks (CMG)	Hispanics (CMG)	Asians (CMG)
Number of samples	5718	1455	2836	122	498	388	51
Average number of nonsynonymous variants per individual	32.332	35.135	60.265	56.156	46.177	45.943	57.176
Fraction of individuals with nonsynonymous variants	1	1	1	1	1	1	1
Average number of predicted deleterious variants per individual	8.132	8.792	12.187	11.984	10.871	10.369	11.176
Fraction of individuals with predicted deleterious variants	1	1	1	1	1	1	1
Average number of ClinVar variants per individual	0.883	0.919	0.527	0.492	0.801	0.675	0.627
Fraction of individuals with ClinVar variants	0.583	0.601	0.397	0.369	0.538	0.487	0.431
Average number of HGMD variants per individual	3.44	3.59	2.346	2.23	3.851	3.423	3.039
Fraction of individuals with HGMD-DM variants	0.965	0.977	0.904	0.926	0.978	0.969	1
Average number of nonsense variants per individuals	0.358	0.402	0.385	0.311	0.416	0.423	0.412
Fraction of individuals with nonsense variants	0.301	0.321	0.316	0.27	0.345	0.338	0.353

These frequencies are reported for: 1) all rare nonsynonymous variants, 2) predicted deleterious variants, 3) reported pathogenic ClinVar variants, 4) HGMD-DM variants, 5) nonsense variants

### Population diversity in ClinVar

A comparison of the number of ClinVar entries among different populations revealed apparent enrichment of variants submitted for European individuals (12,918 out of 36,933 records; Additional file 9). Asians (11,712 records) and Africans (6148 records) are underrepresented in ClinVar, especially if one takes into account the size of the populations in comparison to Europeans.

## Discussion

We sequenced the exomes of 11,068 individuals from a large biracial cohort study and from a study of presumed Mendelian disease that includes individuals from five ethnic groups, and analyzed the frequency of SFs and assessed the autosomal recessive disease allele carrier status. In the 56 ACMG SF genes, the average number of deleterious variants per individual was 0.74, and 51 % of individuals had at least one such variant. There were 642 occurrences of 136 unique SF variants reported in the ClinVar database as pathogenic. There were 1366 reported pathogenic ClinVar variants observed in the list of 1423 autosomal recessive disease genes. We observed 32,213 unique deleterious variants in autosomal recessive disease genes occurring 105,323 times, with an average of 10 such variants per individual. About one-half of one percent of couples are at risk of having an affected offspring for an autosomal recessive disorder based on ClinVar variants reported as pathogenic. Surprisingly, this number is 17.6 % if all observed predicted deleterious alleles in autosomal recessive disorder genes are considered. This forms a likely upper bound for the frequency of at-risk couples as many of these presumed damaging variants may have no effect on protein function or disease risk, emphasizing the future value of experimentally evaluating their potential functional consequences and elucidating their real pathogenicity.

An informative comparison of the SFs observed here with the frequencies reported from previous studies is challenging because the list of genes, variant classification databases and interpretation of clinical significance are evolving. For example, Johnston et al. [31] screened variants in 37 cancer-susceptibility genes, but only 23 of these genes were included in the ACMG list. Berg et al. [32] studied SFs in 2016 genes categorized into “bins” based on clinical utility and validity. One of these bins includes 161 clinically actionable genes, from which 31 genes were used in our study. Dorschner et al. [9] analyzed 114 genes, including 52 from the ACMG recommendation. Despite these differences, it is possible to identify emerging trends.

It is known that individuals of African descent have more nonsynonymous SNVs than individuals from other populations [33] so that one would expect an enrichment of reported pathogenic variants in this group. In fact, we found that the average number of nonsense variants in AAs (0.013) is significantly higher than in EAs (0.007). The analysis of predicted deleterious variants further supports this finding. Similarly, Dorschner et al. [9], who analyzed 500 EAs and 500 AAs, noted that out of five novel likely pathogenic nonsense variants, three were found in AAs. On the other hand, we observe the opposite trend when only previously reported pathogenic variants are considered. For example, the average number of reported pathogenic ClinVar variants in autosomal recessive disease-causing genes is 0.53 in AAs and 0.88 in EAs. Consistent with our observation of the striking deficit of pathogenic variants in AAs, Dorschner et al. [9] reported that only 3 out of 18 pathogenic or likely pathogenic variants were present in individuals of African descent. It was postulated that the most likely explanation for this finding is the underrepresentation of African descent individuals in the clinical genetics literature [9]. Although other hypotheses, such as a European bottleneck, were previously considered to explain the increased number of deleterious variants in Europeans [34], recent studies show no evidence of a higher load of deleterious variants in non-African populations [35]. In this study, we explore the frequency of SF variants in Turkish, Hispanic and Asian populations. In general, we observed that the frequencies of both reported pathogenic and predicted deleterious variants are between the range of the two extremes defined by European and African descent populations.

As previously noted [31], large scale manual curation of variants in the era of massive whole exome or whole genome sequencing to identify clinical pathogenicity is not practical. Therefore, automation of curated databases containing pathogenic variants and better prediction algorithms are each necessary. In addition to the number of HGMD-DM variants, our study reports the frequencies of reported pathogenic variants derived from the ClinVar database. The number of pathogenic variants reported in ClinVar is one-sixth of the number in HGMD-DM [36], and 136 SF ClinVar and 645 SF HGMD-DM variants were identified in this study. Our estimate of the reported pathogenic variant frequency in SF genes (5.6 %) based on ClinVar is slightly higher than the frequencies reported by Dorschner et al. [9] (1.2–3.4 %), who performed additional manual curation. On the other hand, our estimate was based on the annotations provided in ClinVar, which established more conservative and transparent inclusion criteria for pathogenic variants. Although no database is error-free, well-structured repositories not only provide an opportunity to streamline variant filtering and automate the first pass analysis, but also help avoid error-prone subjective decisions intrinsically introduced by manual curation.

In this study, 2171 annotated predicted deleterious nonsynonymous variants in SF genes were not present in either ClinVar or HGMD, and these variants have a significantly lower allele frequency than the variants found in those data resources (Additional file 10). The high frequency of HGMD/ClinVar variants is most likely because they have already been seen in other studies, which increases the prior probability of observing this variant again in a sample from the general population. Having a low MAF is a predictor of variant pathogenicity [37] and a fraction of these rare unclassified variants are likely to be pathogenic. In the case of nonsense variants in SF genes, this fraction can be as high as 39 % based on the observation that 30 out of 76 likely pathogenic nonsense variants in SF genes were not reported in HGMD or ClinVar.

Although reported pathogenic ClinVar variants and novel nonsense variants were found in only a small percentage of individuals, we observed that about half of the individuals in our sample (5674 out of 11,068) have a predicted deleterious nonsynonymous variant in at least one SF gene. We anticipate that with the accelerated pace of gene and pathogenic variant discovery and the growth of commercial clinical sequencing programs [2], the number of ClinVar variants and the amount of additional data evaluating the level of clinical significance of previously reported variants will greatly increase, which will further improve the quality of variant classification. We also observed that African and Asian populations are underrepresented in the ClinVar database (Additional file 9). Therefore, a larger number of submissions from ethnicities other than Europeans will enable more accurate comparison of the burden of pathogenic variants among different populations.

There is growing interest among the public regarding carrier detection for autosomal recessive conditions. Carrier testing is no longer limited to specific ethnic groups having a high frequency of certain conditions or extended families aggregating for a specific condition. Surveys indicate that more than two-thirds of people would like to have their genome sequenced [38]. As the cost of sequencing continues to decline and the ability to interpret the sequence information with respect to health and disease improves, the frequency is likely to continue to increase. Sequence-based carrier tests fall into two subtypes: targeted sequencing of known disease genes and exome or whole genome sequencing. Targeted sequencing lacks the ability to rapidly incorporate newly reported disease genes. In a recent study, 30 % of whole exome-based diagnoses were in genes reported since 2011 [2]. The second category of test holds the most promise because of its comprehensive nature, but suffers from its relatively high cost and the need to catalogue and update potentially large numbers of variants of unknown significance. Formal cost-benefit analyses of these options are limited [39–42], and further studies are needed in this growing molecular diagnostic area.

We estimated that a minimum of 0.5 % of randomly paired individuals are at 1 in 4 risk of having an offspring affected by alleles in a known recessive disease gene. When all predicted deleterious variants are considered, we observed that the fraction of couples being at risk can be as high as 17.6 %. The global prevalence of all monogenic diseases is estimated to be ~10/1000 [43] and autosomal recessive (AR) diseases account for one-third of them [44], implying that frequency of AR disease is ~3/1000. This further suggests that the expected fraction of couples at risk for having an offspring with an AR disorder is ~1.2 % (4 × 3/1000), a value much smaller than 17.6 %. The difference is likely attributable to small effect sizes, incomplete penetrance, subclinical manifestations of Mendelian diseases, or Mendelian forms of common diseases. Similarly, we estimated the percentage of females carrying a reported pathogenic ClinVar variant in X-linked disease genes to be 5 % and those having predicted deleterious variants in the same list of genes to be 33 %. As the number of gene discoveries continues to increase, these proportions will also increase. A comparison of the numbers derived from reported pathogenic variants and the numbers calculated based on predicted deleterious variants (i.e., 0.5 % versus 17.6 % or 5 % versus 33 %) reflects the lower and upper limits of the total number of cases for which potentially pathogenic variants should be evaluated more carefully. While reported pathogenic variants are likely to be included in the primary report of a carrier test, additional deleterious variants of unknown significance can be also considered if additional data are present (e.g., family history) and reevaluated when new information becomes available.

The burden of recessive carrier status has been previously investigated [23, 24, 32, 45, 46]. Lazarin et al. [45] used targeted genotyping and showed that 24 % of individuals are carriers for selected, previously reported recessive alleles in 108 genes. Bell et al. [23] sequenced 437 pediatric recessive disorder genes and obtained somewhat higher estimates (2.8 variants per individual) than that of Lazarin et al. [45]. In our study, we report the average number of variants in 1423 autosomal recessive disease genes to be at least as high as that reported by Bell et al. [23]. We observed that each individual in our study sample is a carrier, on average, of 0.78 reported ClinVar pathogenic variants. Although the false positive rate of available prediction software is still far from excellence (estimated false positive rate of RadialSVM is ~57 % [26]), the average value of ten predicted deleterious variants per individual is a reasonable approximation of the upper limit for the total burden of autosomal recessive carrier status.

## Conclusions

SFs were ascertained in AAs, EAs and five additional populations. Considering the list of 56 SF genes, the expected number of reported pathogenic SFs in each newly sequenced individual is small in comparison with the expected number of predicted deleterious variants. For example, each individual in this study sample has a 1 in 18 (5.6 % of study sample) chance of possessing a previously reported ClinVar variant in one of the 56 ACMG actionable genes. In contrast, when considering all cases with reported pathogenic ClinVar variants, we found that each individual has 1 in 2 risk (51 % of study sample) of having a predicted deleterious nonsynonymous variant in these same genes. Similarly, 0.5 % of couples are at risk of having an affected offspring for an autosomal recessive disorder based on ClinVar variants; this number is 17.6 % considering all observed predicted deleterious alleles in autosomal recessive disorder genes. These data define the upper and lower bound of the frequency of SF findings and carrier detection results. We observed ethnic differences in the frequency of secondary findings and autosomal recessive carrier frequencies. AAs carry, on average, around three nonsynonymous variants in the SF genes, whereas EAs have around two. For carriers of autosomal recessive disease alleles, individuals of European ancestry had the lowest carrier frequency, while individuals of African ancestry had the highest. The highest average number of reported pathogenic variants was found in the European population, but this likely reflects a reporting bias caused by the higher volume of submissions for this widely studied ethnic group (Additional file 9). Clearly, more discovery efforts are needed in non-European ancestry populations.

## Additional files

Additional file 1:
**List of phenotypes in the CMG cohort.**
Additional file 2:
**Describes the procedure of recessive disease gene extraction from MedGen and the procedure we used to compare the number of ClinVar submission for different ethnic groups.**
Additional file 3:
**Venn diagram showing the comparison of the list of autosomal recessive genes generated based on MedGen query to the lists reported in previous studies, i.e., Boone et al. [** 24 **] and Bell et al. [** 23 **].**
Additional file 4:
**The first column of Additional file 4 includes the original list of genes extracted from MedGen that are potentially associated with autosomal recessive disorders.** The second and third columns indicate genes overlapping gene lists from previous studies [23, 24] and the fourth column presents the genes selected for manual curation. The fifth column shows the results of the final selection, i.e., genes overlapping with previous studies or those that passed manual curation. Additional file 5:
**The final list of autosomal recessive genes and conditions obtained from the MedGen database.** It does not include genes that were excluded in the manual curation step (see Methods). Additional file 6:
**The final list of X-linked recessive genes and conditions obtained from the MedGen database.** It does not include genes that were excluded in the manual curation step (see Methods). Additional file 7:
**The first column of Additional file 7 includes the original list of genes extracted from MedGen that are potentially associated with X-linked recessive disorders.** The second column indicates genes that passed manual curation. Additional file 8:
**Presents the distribution and color of the first two principal components relative to a HapMap comparison group for individuals from the CMG cohort.**
Additional file 9:
**Shows the number of ClinVar submissions for European, African, Asians and Hispanics populations.**
Additional file 10:
**Shows differences in MAF between reported pathogenic variants and those not classified in HGMD or ClinVar but predicted to be deleterious in the SF genes (a) or in autosomal recessive disease genes (b).**

## Abbreviations

AA: African American
ACMG: American College of Medical Genetics and Genomics
AR: autosomal recessive
ARIC: Atherosclerosis Risk in Communities
CMG: Center for Mendelian Genomics
DM: disease-causing mutation
EA: European American
ESP: Exome Sequencing Project
HGMD: Human Gene Mutation Database
MAF: minor allele frequency
NMD: nonsense-mediated decay
OMIM: Online Mendelian Inheritance in Man
QC: quality control
SF: secondary finding
SNP: single nucleotide polymorphism
SNV: single nucleotide variant
SVM: support vector machine
