# Supplementary material for: Secondary findings and carrier test frequencies in a large multiethnic sample
Source: Genome Med. 2015 Jun 13;7(1):54. doi: 10.1186/s13073-015-0171-1 (PMC4507324; doi:10.1186/s13073-015-0171-1)
Supplement: Additional file 2: — Describes the procedure of recessive disease gene extraction from MedGen and the procedure we used to compare the number of ClinVar submission for different ethnic groups. [file 13073_2015_171_MOESM2_ESM.doc]

**1. Recessive disease genes extraction from MedGen**

We used the advanced search utility available at the MedGen website (<http://www.ncbi.nlm.nih.gov/medgen/advanced>) with the following query: "autosomal recessive inheritance"[Mode of Inheritance] to extract the list of autosomal recessive disorders. According to MedGen documentation the “mode of inheritance” for each disease was determined based on the data collected in OMIM, ClinVar and GTR. Next we used an in-house python script that iterates through all of MedGen identifiers obtained in the previous step and downloads the data associated with these identifiers from the URLs generated in the following format: [http://www.ncbi.nlm.nih.gov/medgen/[MedgenID](http://www.ncbi.nlm.nih.gov/medgen/%5BMedgenID)]. Finally, we used the pyquery python library to parse the downloaded html pages and extracted the complete list of genes associated with each disease.

If the disease had multiple modes of inheritance we still extracted all the genes associated with this record. Since this may lead to some incorrect assignments we applied additional curation procedure which includes: (i) the intersection of genes extracted from MedGen with previously described lists of autosomal recessive genes and (ii) the manual inspection of the OMIM entries for genes extracted from MedGen but not found in previous studies.

**2. Ethnicity breakdown in ClinVar submissions**

Using "ClinVarFullRelease_00-latest.xml" file downloaded from ftp://ftp.ncbi.nlm.nih.gov/pub/clinvar/xml/ on February 16, 2015, we extracted 49,598 of the records with <Ethnicity>...</Ethnicity> tag. Since the content of the tag is provided in the form of free text, rather than fixed list of different ethnic groups, we used regular expression to categorize these entries into 4 populations: "Europeans", "Africans", "Hispanics" and "Asians". For example, to determine which records corresponds to Europeans we used the following regex: "europ|cauca|greek|dutch|english|german|austrian|french|polish|scotish|scottish|galician|italian|latvian". Using this approach we categorized 42,845 out of 49,598 records. The large fraction of non-categorized records (5,376 out of 6,753) were annotated as "Whole_cohort". Manual inspection of the remaining 1,377 records revealed that the majority of these records are associated either with Middle Eastern or South American ethnicities. After excluding 5,912 records with multiple categories assigned in the first step (e.g. "african_european" was assigned to "European" and "African" category) we obtained 36,933 records each categorized into one of 4 populations.
