# Supplementary figures and images for: Secondary findings and carrier test frequencies in a large multiethnic sample
Source: Genome Med. 2015 Jun 13;7(1):54. doi: 10.1186/s13073-015-0171-1 (PMC4507324; doi:10.1186/s13073-015-0171-1)

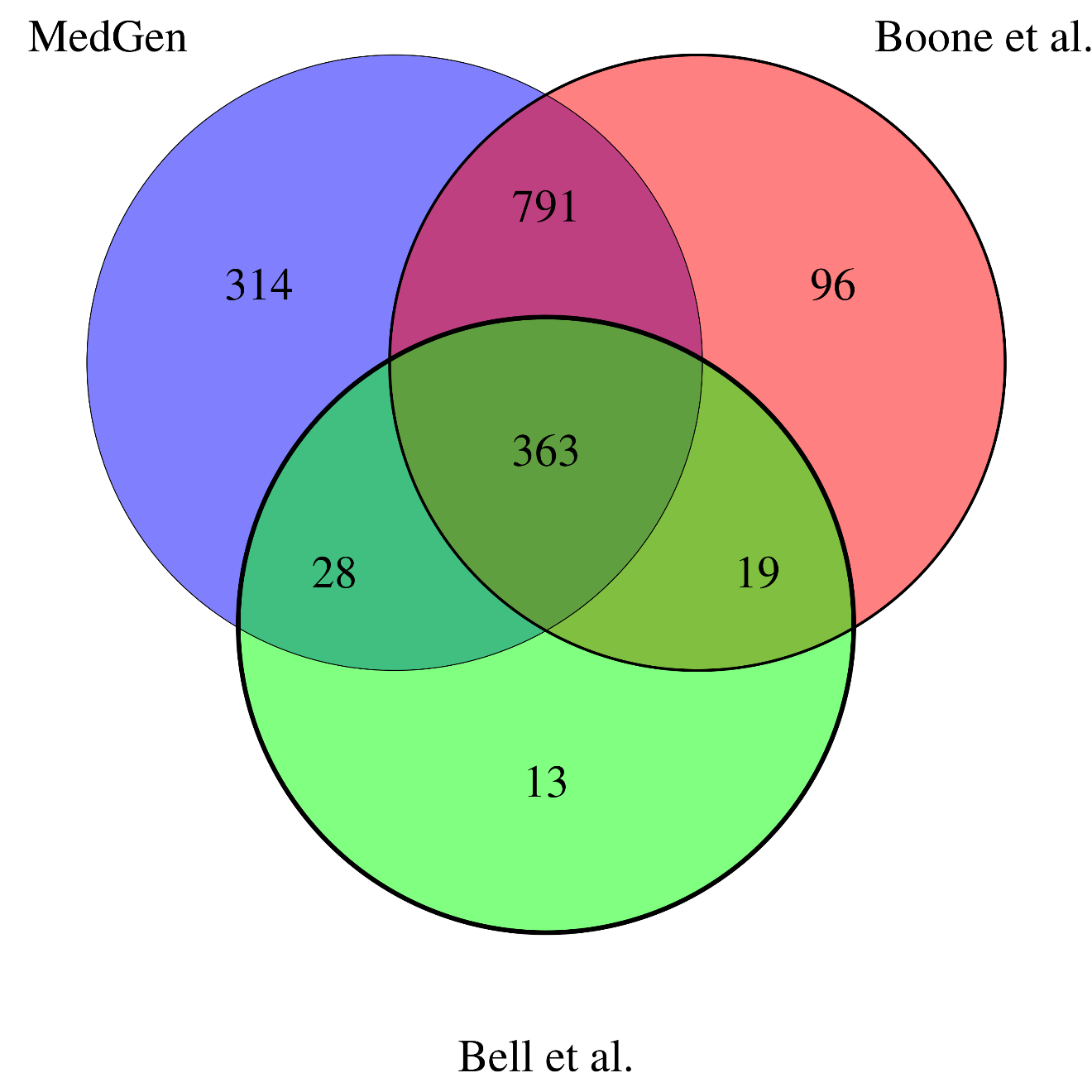

Supplement: Additional file 3: — Venn diagram showing the comparison of the list of autosomal recessive genes generated based on MedGen query to the lists reported in previous studies, i.e., Boone et al. [ 24 ] and Bell et al. [ 23 ]. [file 13073_2015_171_MOESM3_ESM.png]

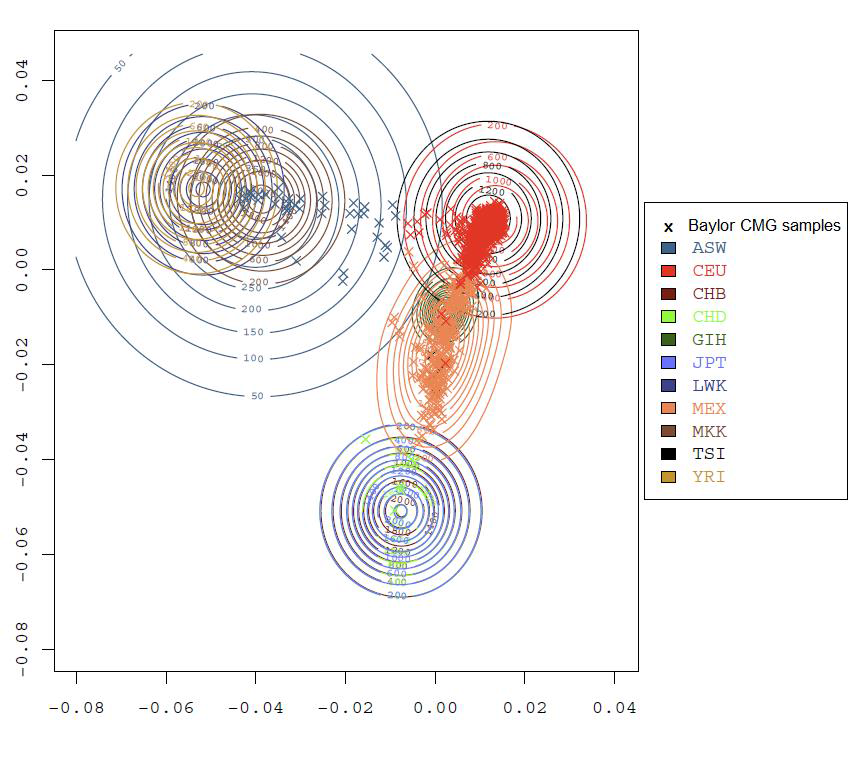

Supplement: Additional file 8: — Presents the distribution and color of the first two principal components relative to a HapMap comparison group for individuals from the CMG cohort. [file 13073_2015_171_MOESM8_ESM.png]

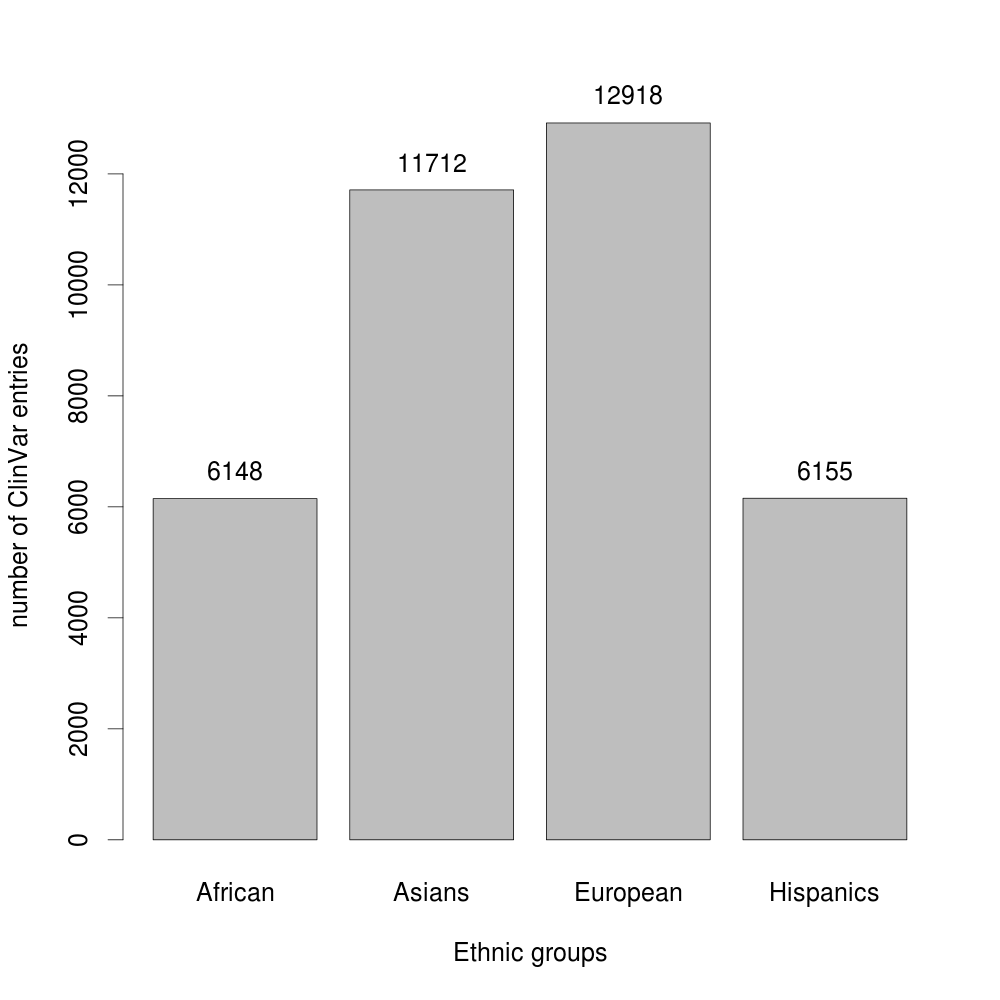

Supplement: Additional file 9: — Shows the number of ClinVar submissions for European, African, Asians and Hispanics populations. [file 13073_2015_171_MOESM9_ESM.png]

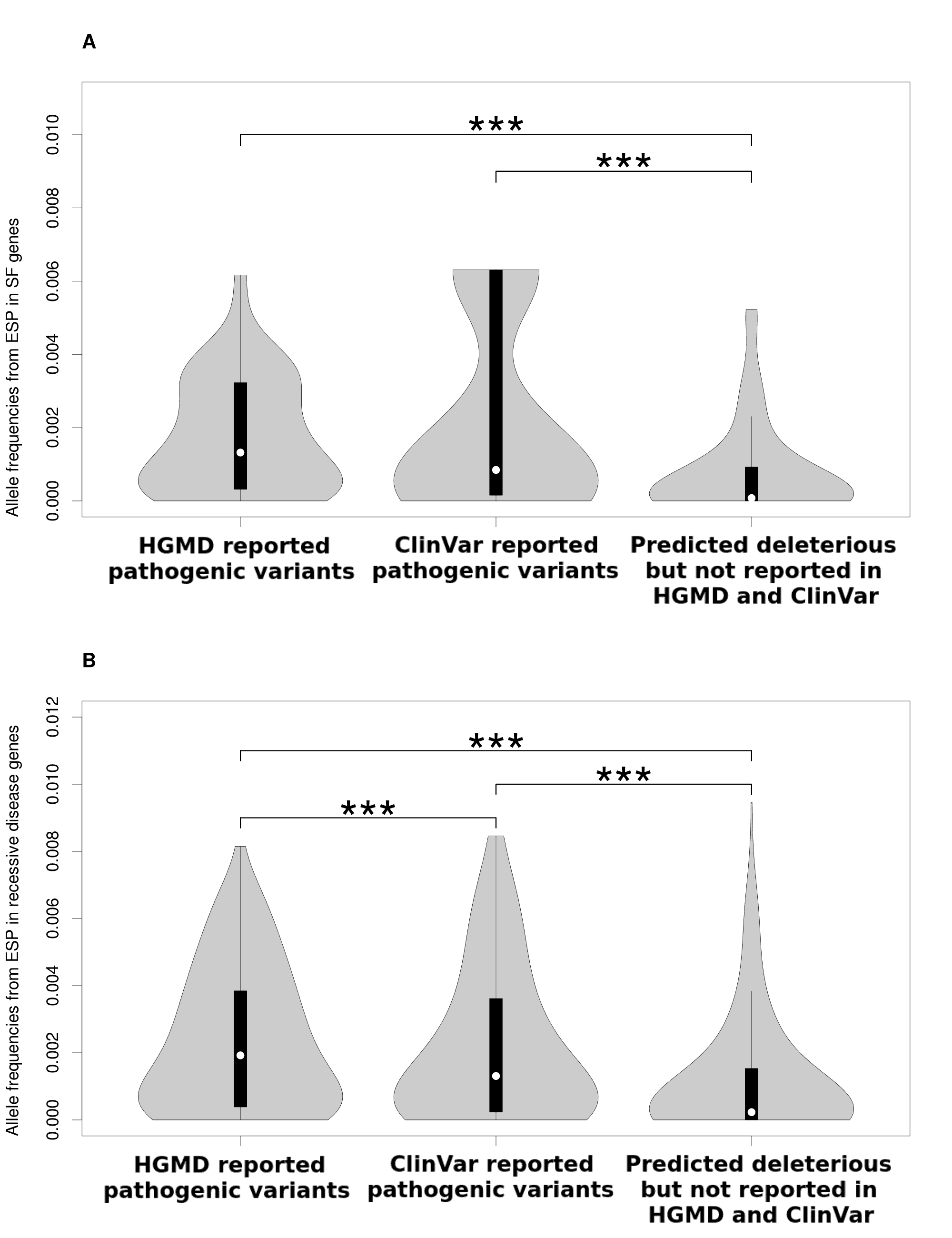

Supplement: Additional file 10: — Shows differences in MAF between reported pathogenic variants and those not classified in HGMD or ClinVar but predicted to be deleterious in the SF genes (a) or in autosomal recessive disease genes (b). [file 13073_2015_171_MOESM10_ESM.png]
